# Anti-Atherogenic Mechanisms and Therapies

**DOI:** 10.1007/s11883-025-01324-9

**Published:** 2025-08-20

**Authors:** Alex Hudson, Oluwakemi O. Igiehon, Matthew D. Woolard, Arif Yurdagul

**Affiliations:** 1 Department of Molecular and Cellular Physiology, LSU Health Sciences Center at Shreveport, Shreveport, LA USA; 2 Department of Microbiology and Immunology, LSU Health Sciences Center at Shreveport, Shreveport, LA USA; 3 Center for Applied Immunology and Pathological Processes, LSU Health Sciences Center at Shreveport, Shreveport, LA USA; 4 Department of Pathology and Translational Pathobiology, LSU Health Sciences Center at Shreveport, Shreveport, LA USA

**Keywords:** Atherosclerosis, Efferocytosis, Macrophage metabolism, Efferotabolism, Inflammation resolution, Pro-resolving mediators

## Abstract

**Purpose of Review:**

This review examines anti-atherogenic mechanisms and the crucial role of efferocytosis in promoting inflammation resolution, with a focus on innovative, resolution-based therapeutic strategies that aim to restore vascular homeostasis and mitigate atherosclerosis progression.

**Recent Findings:**

Atherosclerosis, a chronic inflammatory condition, is exacerbated by impaired efferocytosis, which contributes to plaque instability and the expansion of the necrotic core. Advanced molecular and cellular profiling has revealed diverse macrophage populations and their metabolic adaptations during efferocytosis, which drive the production of resolving mediators essential for tissue repair. Dysregulated signaling and metabolic pathways disrupt the efficient clearance of apoptotic cells, exacerbating inflammation. Molecular regulators, such as microRNAs, further impact efferocytosis, governing cardiovascular outcomes. Resolution-based therapies, including specialized pro-resolving mediators, peptides, and metabolites, enhance the successive clearance of apoptotic cells while maintaining host immune function, offering advantages over traditional immunosuppressive approaches. Additionally, vaccines targeting disease-specific antigens show promise in eliciting protective immune responses that can help ameliorate atherosclerosis.

**Summary:**

Efferocytosis is a key regulator of inflammation resolution in atherosclerosis, linking macrophage metabolism to plaque stability. Its disruption drives disease progression, but emerging therapies targeting resolution pathways, metabolic reprogramming, and immune modulation hold the potential for effective interventions. Advances in profiling technologies and targeted delivery systems will address translational challenges, paving the way for precision medicine in treating atherosclerotic cardiovascular disease.

## Introduction

Atherosclerosis is a chronic inflammatory disease initiated by the subendothelial retention of apolipoprotein B-containing lipoproteins and sustained by dysregulated immune responses [1, 2]. While historically considered a lipid storage disorder, atherosclerosis is now recognized as a metabolically driven inflammatory disease characterized by unresolved inflammation, defective clearance of apoptotic cells (ACs), and progressive tissue remodeling [3–8]. Central to these processes are macrophages, which participate in lipid uptake, cytokine production, and efferocytosis, controlling the balance between inflammation and resolution within the plaque microenvironment [1–3, 9]. In early atherogenesis, efficient AC clearance and pro-resolving signaling promote repair and maintain homeostasis [7, 10]. However, these reparative programs become progressively impaired, driving features of plaque instability, including necrotic core expansion and fibrous cap thinning [10–12].

Emerging evidence suggests that efferocytosis serves as a metabolic checkpoint, reprogramming macrophage bioenergetics to modulate inflammatory signaling, antigen presentation, and tissue repair [5, 8, 13]. These insights position immunometabolism and efferotabolism as a key axis linking innate immunity to plaque stability [5, 14]. In this review, we examine how efferocytosis influences macrophage metabolism, how this rewiring regulates resolution pathways, and how these processes are disrupted in atherosclerosis. Emphasis is placed on emerging therapies promoting inflammation resolution while preserving host defense. Advancing these approaches provides a foundation for next-generation interventions aimed at restoring vascular homeostasis to a disease marked by silent progression and adverse clinical outcomes.

## Cellular and Molecular Drivers of Chronic Inflammation in Atherogenesis

While lipid accumulation initiates plaque formation, sustained engagement of innate and adaptive immune responses drives chronic inflammation and atherogenesis [1, 9]. Advances in single-cell and spatial profiling have identified diverse immune cell subsets within plaques, uncovering key cellular and molecular programs perpetuating inflammation [15–17]. This section highlights macrophages, T cells, and B cells, whose interactions influence chronic inflammation and its resolution.

### Macrophages

Single-cell RNA sequencing has expanded our understanding of macrophage diversity beyond traditional foam cells, revealing subsets such as resident-like (LYVE1⁺), pro-inflammatory (CD86⁺, iNOS⁺), anti-inflammatory (CD206⁺, Arg1⁺), and lipid-sensitive Trem2⁺ cells [18]. These phenotypes arise from local stimuli, including oxidized lipids, cytokines, and their interactions with dead cells (e.g., apoptosis, necrosis, necroptosis, pyroptosis, and ferroptosis) that shape macrophage functions. Oxidized LDL consists of LDL particles with peroxidized phospholipids, oxidized cholesterol esters, and modified ApoB protein components, each contributing to its pro-inflammatory and immunogenic properties [19–22]. OxLDL forms in the subendothelial space when retained LDL particles undergo oxidative modifications catalyzed by reactive oxygen species, myeloperoxidase, lipoxygenases, and other oxidant enzymes released by activated endothelial cells, macrophages, and neutrophils [19–22]. These modifications generate a spectrum of oxidized lipids and epitopes that drive inflammation and immune activation. In parallel, oxLDL and cholesterol crystals activate inflammatory pathways in vascular cells, including the NLRP3 inflammasome, leading to IL-1β-mediated inflammation [23]. Dedifferentiation of vSMCs and damage-associated molecular patterns (DAMP) release may further amplify inflammatory responses in the plaque environment [24]. However, during the early stages of atherosclerosis, interactions with ACs and subsequent efferocytosis elicit pro-resolving responses from recruited and resident macrophages [25, 26]. As atherosclerosis advances, recruited monocytes differentiate into macrophages and produce reactive oxygen species, reactive nitrogen species, and inflammatory cytokines through the NLRP3 inflammasome and TLR signaling [27, 28]. These pathways reinforce a self-sustaining inflammatory loop [28], which can be further stabilized through epigenetic reprogramming that establishes innate immune memory [29]. While foamy macrophages were historically viewed as detrimental, recent studies have redefined this perspective by identifying a Trem2⁺ foam cell subset with enhanced lipid handling, cholesterol efflux, and survival capacity [30, 31]. Trem2 agonism via AL002a improves these properties and enhances plaque stability by reducing necrotic core formation and increasing fibrous cap thickness [31]. Macrophage subsets also interact extensively with T and B cells within the plaque microenvironment, shaping local immunity and disease outcomes.

### T Cells

Adaptive immunity plays a crucial role in directing macrophage function. Lesional CD4⁺ T cells differentiate into Th1 and Th17 subsets, producing IFN-γ and IL-17, respectively, polarizing macrophages toward a pro-inflammatory phenotype marked by ROS production and inflammasome activation [32–34]. These responses are antigen-driven, with clonally expanded T cells specific for oxidized LDL and ApoB neoepitopes commonly found in human and murine plaques [32]. Regulatory T cells (Tregs) counterbalance these effects by promoting macrophage-mediated inflammation resolution. Through the secretion of IL-10 and IL-13, Tregs enhance MerTK-dependent efferocytosis and limit the expansion of the necrotic core [35, 36]. However, in advanced atherosclerosis, Treg numbers decline, and the remaining cells become functionally impaired [37]. Interestingly, exposure to atherogenic stimuli, such as hypoxia, oxidized lipids, and inflammatory cytokines, can reduce FoxP3 expression, converting Tregs into IFN-γ⁺ or IL-17⁺ effector-like cells that further exacerbate inflammation [38, 39]. This shift disrupts the balance between effector and regulatory T cells, sustaining cytokine-driven monocyte recruitment and macrophage activation [40, 41].

### B Cells

B cells are now recognized as key modulators of atherosclerosis through both antibody production and immune regulation. B1 cells produce natural IgM antibodies, such as T15/E06, that bind oxidized phospholipids on oxLDL and ACs, facilitating their clearance [42]. These IgM-opsonized ACs are recognized by complement C1q and cleared via MerTK, promoting a pro-resolving IL-10⁺/TGFβ⁺ macrophage phenotype and enhancing plaque stability [43]. In contrast, follicular B2 cells support atherogenesis by presenting antigens to CD4⁺ T cells, promoting Th1 expansion in the lymph nodes, which activates lesional macrophages [44, 45]. B2 cells secret TNFα and IL-6, which may contribute to macrophage activation and inflammasome priming within the atherosclerotic milieu [45]. Additionally, class-switched IgG1/IgG2 antibodies from B2 cells opsonize oxLDL and engage Fcγ receptors on macrophages, promoting ROS production and cytokine release [46]. Regulatory B cells (Bregs) counteract these effects by producing IL-10 and promoting TGFβ signaling and efferocytosis, thereby limiting the growth of the necrotic core and inflammation [47, 48].

The functional balance between protective and pathogenic B cell subsets strongly influences lesion development and plaque stability. Shifts in antibody isotype and subclass further correlate with instability, reflecting the evolving humoral response to chronic antigen exposure. Crosstalk among B cells, T cells, and macrophages amplifies inflammation and can impair resolution. However, recent studies suggest that vaccine-based strategies may harness protective B and T cell responses to promote macrophage resolution programs, a concept explored further in later sections [49].

## Failure to Resolve: Impaired Efferocytosis in Atherosclerosis Progression

**Fig. 1:**
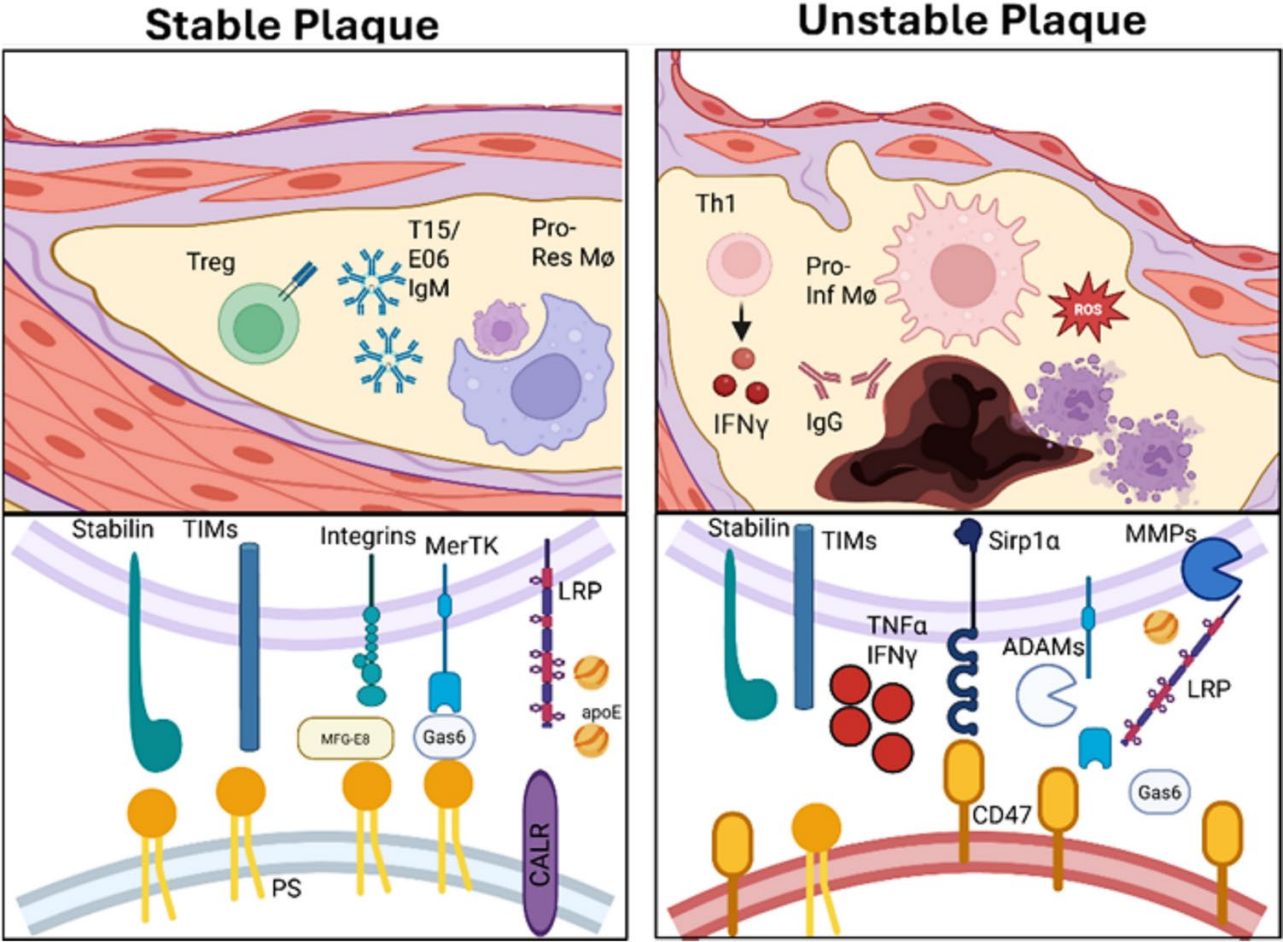
Immune regulation of efferocytosis in stable and unstable atherosclerotic plaques. In stable plaques, effective efferocytosis by pro-resolving macrophages preserves plaque integrity. This process is supported by regulatory T cells, protective natural IgM antibodies, and macrophage subsets expressing apoptotic cell (AC) receptors. ACs are recognized directly via phosphatidylserine-binding receptors such as TIMs and Stabilins, or indirectly via bridging molecules (e.g., MFG-E8, GAS6, Protein S) that link phosphatidylserine or calreticulin to MerTK, integrins (αvβ3/αvβ5), and LRP1. In contrast, unstable plaques in advanced disease are characterized by a shift toward pro-inflammatory immune responses, including Th1 and Th17 cells, IgG-switched antibodies, and macrophages that generate reactive oxygen species. These inflammatory cues impair the clearance of dead cells through multiple mechanisms: proteolytic cleavage of MerTK and LRP1 by ADAMs and MMPs; downregulation of bridging molecules such as GAS6 and MFG-E8; and increased expression of “don’t-eat-me” signals such as CD47, which inhibit phagocytic uptake via SIRPα signaling. Impaired clearance promotes post-apoptotic necrosis, expansion of the necrotic core, and thinning of the fibrous cap, thereby driving plaque instability

While sustained inflammation drives plaque expansion, resolution efforts aimed at restoring balance are also mounted. However, a shift towards pro-inflammatory macrophage polarization occurs as atherosclerosis progresses, lowering AC clearance as fewer pro-resolving macrophages are available [50–52]. As ACs accumulate during atherosclerosis, lesional macrophages become overburdened and fail to clear them efficiently, resulting in necrotic core expansion and plaque instability (Fig. 1) [6]. Therefore, understanding the various cellular and molecular mechanisms that drive impaired efferocytosis in atherosclerosis is crucial for identifying new therapeutic targets for the disease.

Many “find-me”, “eat-me”, and “don’t-eat-me” signals are altered as atherosclerosis advances. ACs recruit phagocytes to sites of necrosis by releasing chemotactic “find-me” molecules, including lysophosphatidylcholine (LPC), fractalkine (CX3CL1), sphingosine-1-phosphate, and nucleotides (ATP/UTP) [6, 52]. LPC levels become dysregulated in patients with atherosclerosis [53, 54], and it has been proposed that oxLDL-derived LPC competes with AC-derived LPC, potentially interfering with phagocyte recruitment. Additionally, the “eat-me” signal calreticulin is decreased in lesional macrophages in advanced plaques [55], and CD47, a “don’t-eat-me” signal, is abundantly expressed on cells undergoing necroptosis [56, 57], tipping the balance towards defective efferocytosis and an accumulation of necrotic debris. Mechanistically, CD47 on necroptotic cells engages with SIRPα on macrophages, delivering an inhibitory signal that blocks engulfment. Therapeutic blockade of the CD47/SIRPα axis using blocking antibodies increases the clearance of dead cells and reduces experimental atherosclerosis [58].

The degradation, downregulation, or dysfunction of lesional macrophage AC receptors and bridging molecules contribute to defective efferocytosis (Fig. 1). These receptors are essential for the recognition of phosphatidylserine, the dominant “eat-me” signal displayed on ACs [59]. Several receptors, including the TIM family (TIM1, TIM3, and TIM4), Stabilin-1, Stabilin-2, brain-specific angiogenesis inhibitor 1 (BAI1), and the CD300 family of receptors, can bind phosphatidylserine directly. In contrast, TAM receptors (Tyro3, Axl, and MerTK), integrins (such as αvβ3 and αvβ5), and the scavenger receptor CD36 rely on bridging molecules, such as GAS6, Protein S, and MFG-E8, to mediate recognition and engulfment of ACs [60–62]. During atherosclerosis, oxidative stress can lead to ADAM17 production and proteolytic cleavage of MerTK, generating a soluble form that acts as a decoy receptor for the MerTK ligand GAS6 and thereby blocking efferocytosis [63, 64]. CD147, which is upregulated in atherosclerotic plaques, further impairs efferocytosis by suppressing GAS6 expression through STAT6 inactivation in macrophages [65]. Deletion of LRP1 in macrophages impairs AC clearance and promotes necrotic core expansion, contributing to plaque progression [66–69].

As the lesional lipid burden increases with disease progression, the accumulation of oxLDL and MerTK cleavage impairs efferocytosis by macrophages, contributing to necrotic core expansion [62–64]. Furthermore, oxLDL induces membrane lipid redistribution in macrophages, resulting in distorted actin cytoskeleton remodeling and polymerization, as well as altered lysosomal dynamics, which may interfere with migration and phagocytosis [70]. Furthermore, pro-inflammatory cholesterol crystals induce membrane rupture and necrosis in phagocytes by sequestering cholesterol from the plasma membrane [71]. Lipid overload also disrupts the function and intracellular signaling pathways of macrophage organelles. For instance, ER stress, marked by ER membrane disruption, ROS generation, dysregulation of intracellular calcium homeostasis and signaling, and protein misfolding, can be induced by lipid overload, leading to defective efferocytosis, heightened apoptosis, and foam cell formation, thereby promoting atherosclerosis progression [72, 73].

Efferocytosis can be disrupted by microRNA-mediated repression of key molecules involved in AC recognition, uptake, and processing. The atherogenic microRNA miR-34a impairs efferocytosis by downregulating AXL and SIRT1, both of which are important for AC clearance [66–68]. Additionally, oxLDL-induced miR-155-5p contributes to a feedforward loop of cholesterol accumulation, which further impairs efferocytosis [69]. miRNA-342-5p suppresses efferocytosis indirectly by upregulating miR-155 through inhibition of its negative regulator Akt, while miR-33 inhibits efferocytosis by ameliorating macrophage autophagy [26]. These dysregulated miRNA pathways collectively impair macrophage efferocytosis at multiple levels, contributing to defective clearance of ACs in atherosclerotic lesions.

## Macrophage Metabolism in Atherosclerosis

Macrophages are metabolically adaptable cells capable of reprogramming their metabolic pathways in response to environmental cues, leading to changes in polarization states that influence the initiation, progression, or regression of atherosclerosis. In early atherogenesis, oxLDL promotes the recruitment of circulating monocytes and their differentiation into pro-inflammatory macrophages. There is also a metabolic shift toward aerobic glycolysis, driven in part by HIF1α- and mTOR signaling, which support increased ATP production, pro-inflammatory cytokine expression, and ROS generation, thereby sustaining local inflammation and promoting further immune cell recruitment [74].

Scavenger receptor-mediated oxLDL uptake through CD36, SR-A, or LOX1 leads to intracellular accumulation of free cholesterol and cholesterol esters, accompanied by impaired cholesterol trafficking and efflux, culminating in foam cell formation [75]. Specifically, acyl-CoA:cholesterol acyltransferase 1 (ACAT1) esterifies cholesterol for storage in lipid droplets, whereas neutral cholesterol ester hydrolase and lysosomal acid lipase hydrolyze stored cholesterol esters into free cholesterol that can be exported by ABCA1 and ABCG1 [76]. Notably, blocking cholesterol efflux through ABCA1 and ABCG1 deletion is atherogenic [77]. Additionally, ceramide levels are elevated in both plasma and atherosclerotic plaques [78, 79], where they contribute to foam cell formation by inhibiting macrophage actin polymerization and digestion of aggregated LDL [80]. Impaired LDL catabolism, excessive cholesterol uptake, aberrant esterification, and defective efflux collectively promote lipid accumulation in macrophages. This lipid overload induces cytotoxicity and oxidative stress, impairing fatty acid oxidation (FAO) and OXPHOS [81]. Autophagy, a critical cellular recycling mechanism that supports cholesterol processing and efflux, is also disrupted in macrophages during atherosclerosis [82]. Notably, deletion of the key autophagy gene *Atg5* in macrophages increases oxidative stress, apoptosis, and necrosis [82], highlighting the protective roles of autophagy in maintaining plaque stability.

Amino acid metabolism is a critical regulator of macrophage function during atherosclerosis. L-arginine is metabolized by two competing enzymes, inducible nitric oxide synthase (iNOS) and arginase 1 (ARG1), which drive macrophage polarization toward distinct phenotypes [34]. iNOS activity promotes the production of nitric oxide (NO), supporting a pro-inflammatory phenotype. In contrast, ARG1 activity facilitates the generation of ornithine and polyamines, contributing to a pro-resolving phenotype associated with tissue repair and resolution of inflammation. Macrophages synthesize putrescine from AC-derived arginine and ornithine to sustain efferocytosis, MerTK expression, IL-10 production, inflammation resolution, and, ultimately, atherosclerosis regression [83, 84]. Additionally, nanoparticle-delivered spermine attenuates atherosclerosis progression in *Apoe*^−/−^ mice by limiting macrophage ferroptosis [85]. Similarly, spermidine promotes pro-resolving macrophage polarization by activating AMPK through mitochondrial superoxide production [86]. The branched-chain amino acids (BCAAs) valine, leucine, and isoleucine can support pro-resolving macrophage polarization through their catabolism. However, excessive dietary BCAA intake may instead promote atherosclerosis progression by shifting macrophages toward a pro-inflammatory phenotype, in part through increased mitochondrial-to-nuclear HMGB1 signaling [87–89].

## Metabolic Reprogramming after Apoptotic Cell Clearance

Efferocytosis extends beyond the mere clearance of ACs, it initiates a metabolic program that supports macrophage-mediated inflammation resolution, tissue remodeling, and vascular homeostasis [5, 7]. Following engulfment, macrophages undergo coordinated metabolic rewiring in response to changes in nutrient availability, receptor engagement, and intracellular signaling cascades[5]. This reprogramming integrates glycolysis, FAO, OXPHOS, and amino acid metabolism to align cellular metabolism with pro-resolving functions [8]. AC-derived fatty acids fuel β-oxidation, generating acetyl-CoA to drive mitochondrial ATP production [90]. The shift toward mitochondrial respiration coincides with transcriptional activation of PPARs and LXRs, nuclear receptors that are responsive to oxidized phospholipids and sterol derivatives released during efferocytosis [91–94]. PPARs promote FAO, while LXRs upregulate the cholesterol transporters ABCA1 and ABCG1, enhancing cholesterol efflux, protecting against foam cell formation, and blocking ROS-induced macrophage apoptosis during efferocytosis [95–98]. Together, these metabolic shifts promote the production of pro-resolving mediators [3, 5, 6, 10, 99].

Additionally, efferotabolism influences redox balance and epigenetic regulation. Increased electron transport and OXPHOS activate NAD^+^-dependent enzymes such as SIRT1, which deacetylates NF-κB and promotes PBX1 activation [90, 100, 101]. Together, these transcriptional changes suppress pro-inflammatory gene expression while enhancing IL-10 production, sustaining a feedforward loop that reinforces OXPHOS and resolution signaling. In contrast, macrophages in advanced plaques often exhibit mitochondrial dysfunction, reduced FAO capacity, and disrupted NAD^+^/NADH balance, features associated with defective efferocytosis and necrotic core expansion [5, 56, 102, 103]. Apoptotic cell uptake also reprograms amino acid metabolism. Arginine is shunted through ARG1 to produce ornithine and polyamines, which support extracellular matrix remodeling and collagen deposition during repair [83, 84]. AC-derived methionine fuels methylation reactions that lead to epigenetic modifications, further shaping gene expression [104]. Together, these pathways may constitute a form of “metabolic imprinting” in macrophages, coupling efferocytosis to sustained changes in functional identity via epigenetic remodeling. Additionally, macrophage efferocytosis induces an indoleamine 2,3-dioxygenase-1 (IDO1)-dependent tryptophan metabolic pathway that enhances IL-10 production and promotes continual efferocytosis, thereby facilitating inflammation resolution [105]. Taken together, efferotabolism represents a key metabolic node that enables inflammation resolution, governs macrophage plasticity, and integrates lipid and amino acid metabolism along with balancing redox status. Failure of this axis is a defining feature of advanced atherosclerotic plaques and an attractive target for next-generation therapies.

## Immunosuppressive versus Resolution-Directed Therapies

Exploratory anti-inflammatory strategies for atherosclerosis have primarily relied on immunosuppressive agents that broadly attenuate immune responses. Although effective in autoimmune and rheumatologic diseases these agents carry systemic risks, including impaired host defense. The Cardiovascular Inflammation Reduction Trial (CIRT), which investigated the use of low-dose methotrexate in patients who experienced a previous myocardial infarction or with stable coronary disease and diabetes or metabolic syndrome, failed to reduce IL-1β, IL-6, or C-reactive protein levels and showed no improvement in cardiovascular outcomes [106]. In contrast, the Canakinumab Anti-inflammatory Thrombosis Outcomes Study (CANTOS) provided proof of concept that selective anti-cytokine therapy can reduce cardiovascular risk. IL-1β inhibition with canakinumab significantly lowered rates of major adverse cardiovascular events (MACE) independent of lipid-lowering, validating the inflammation hypothesis of atherosclerosis [107]. However, this benefit came with a modest increase in fatal infections, highlighting the tradeoffs of broadly dampening innate immune signaling without engaging tissue repair mechanisms. More recently, trials of low-dose colchicine, a broad but low-grade anti-inflammatory agent, have further advanced inflammation-targeted therapy. The COLCOT and LoDoCo2 trials demonstrated that colchicine reduces cardiovascular events in patients with recent myocardial infarction or chronic coronary syndrome, respectively [108, 109]. Although its precise mechanisms remain incompletely defined, colchicine is considered to inhibit NLRP3 inflammasome assembly, IL-1β maturation, and leukocyte recruitment [110–112]. These findings reinforce the clinical value of targeting inflammation in atherosclerosis. However, they also underscore a key limitation: current anti-inflammatory approaches primarily blunt upstream cytokine signaling without engaging resolution pathways.

Resolution-based therapies offer a mechanistically distinct strategy (Table 1) [58, 83, 84, 113–121]. Rather than suppressing inflammation, they reactivate endogenous programs that terminate inflammation, clear apoptotic debris, and promote tissue repair [4, 10, 122]. Central to this are macrophage-mediated efferocytosis and the production of SPMs. These bioactive lipids, including resolvins, lipoxins, maresins, and protectins, are derived from omega-3 and arachidonic acid and act through G-protein–coupled receptors to suppress pro-inflammatory signaling, limit leukocyte recruitment, and stimulate tissue remodeling [10, 122]. Unlike conventional anti-inflammatories, SPMs promote resolution without compromising host defense. Importantly, preclinical studies demonstrate that SPMs such as Resolvin D1, Resolvin D2, and Maresin 1 enhance efferocytosis, reduce necrotic core area, stabilize plaques, and improve vascular function (Table 1) [117, 123, 124]. Importantly, SPM biosynthesis is impaired in advanced lesions, supporting the rationale for exogenous supplementation or restoration of endogenous production[119, 125]. Synthetic analogs with enhanced metabolic stability and increased bioavailability are currently under development. Additional resolution-focused strategies include augmenting intracellular NAD⁺ to boost SIRT1 activity, delivering itaconate analogs to suppress inflammatory metabolism, restoring efferocytosis by stabilizing receptors such as MerTK, and supplying polyamines [83–85, 90, 121, 126]. An alternative approach, which does not directly target resolution pathways but may create a permissive environment for resolution, is vaccination. Vaccine-based immunotherapy against atherosclerosis-relevant antigens, such as ApoB-derived peptides, has shown promise in preclinical models by expanding FoxP3⁺ regulatory T cells and IL-10-producing B cells [49, 127–131].

**Table 1:** Examples of preclinical pro-resolving therapeutic agents. Examples of preclinical pro-resolving therapeutic agents, including SPMs, peptides, polyamines, and other resolution-directed strategies shown to enhance efferocytosis, reduce inflammation, and promote features of plaque stability in experimental models of atherosclerosis

Therapeutic Strategy	Type	Model	Intervention/Effect	Reference
Annexin A1 (Ac2-26)	Peptide/Theranostics	Ldlr^-/−^ mice, established atherosclerosis	Nanoparticle delivery decreased oxidative stress, necrosis, increased fibrous cap, collagen synthesis, IL-10 expression	[113]
Aspirin-triggered Lipoxin A4 (AT- LXA4)	SPM	Apoe^−/−^ mice	Osmotic pump administration decreased plaque size	[114]
CaMKlly siRNA nanoparticles	Theranostics	Ldlr^−/−^ mice	siRNA targeting CaMKlly in macrophages improved efferocytosis, reduced plaque necrosis, and increased features of plaque stability	[115]
CD47-blocking antibodies	Antibody therapy	Ldlr^−/−^, Apoe^−/−^ mice	Restored efferocytosis, reduced AC accumulation, and ameliorated atherosclerosis	[58]
IL-10	Cytokine/Theranostics	Ldlr^−/−^ mice	Nanoparticle delivery increased fibrous cap thickness, efferocytosis, and decreased oxidative stress and necrosis	[116]
Maresin 1 and Resolvin D2	SPMs	Apoe^−/−^ mice	Combined administration halted necrosis	[117]
MerTK protein delivery via hybrid membrane nanovesicles	Theranostics	Diabetic Apoe^−/−^ mice	Enhanced efferocytosis and attenuated atherosclerosis	[118]
Putrescine	Polyamine	Ldlr^−/−^ mice	Enhanced continual efferocytosis and increased IL-10 production	[83, 84]
Resolvin D1	SPM	Ldlr^−/−^mice with established atherosclerosis	Increased SPM/leukotriene ratio, enhanced efferocytosis, and decreased necrosis	[119]
Resolvin E1	SPM	Hypercholesterolemic rabbits	Oral/Topical administration decreased atherogenesis	[120]
Spermidine	Polyamine	Apoe^−/−^mice	Reduced necrotic core area and lipid accumulation via autophagy-driven cholesterol efflux	[121]

## Conclusions and Future Directions

Cutting-edge tools such as spatial transcriptomics, mass spectrometry imaging, and single-cell multi-omics are uncovering distinct macrophage subsets and their microenvironmental cues within the plaque [15–17]. These approaches have revealed heterogeneity in efferocytic capacity, metabolic programming, and inflammatory phenotypes, identifying potential therapeutic targets. Integration of these datasets with machine learning frameworks promises to generate predictive models of plaque behavior, therapeutic responses, and clinical outcomes. Future therapies will likely focus on restoring resolution circuits through targeted metabolic reprogramming, re-engagement of efferocytosis, and delivery of pro-resolving mediators. Precision delivery platforms, including ligand-targeted nanoparticles and engineered extracellular vesicles, may enable cell-specific delivery of these therapies while minimizing off-target effects. However, the complexity of human atherosclerosis, characterized by long disease latency and inter-individual variability, poses significant translational challenges. Robust biomarkers of resolution activity, along with tools to monitor efferocytosis and macrophage metabolism in humans, will be crucial for successful implementation. Ultimately, recognizing atherosclerosis as a disease of failed resolution rather than immune hyperactivity has begun to expand the therapeutic landscape, opening new avenues for targeted, pro-resolving interventions. Continued investment in mechanistic discovery, delivery technologies, and integrative analyses will be essential to translate these strategies into next-generation therapies for atherosclerosis.

## Key References

- Ampomah PB, Cai B, Sukka SR, Gerlach BD, Yurdagul A, Jr., Wang X, et al. Macrophages use apoptotic cell-derived methionine and DNMT3A during efferocytosis to promote tissue resolution. Nat Metab. 2022;4(4):444–57. 10.1038/s42255/022/00551-7.
  - ⚬This study discovered that macrophages metabolize apoptotic cell-derived methionine via DNMT3A to support epigenetic reprogramming and resolution of inflammation.
- Sukka SR, Ampomah PB, Darville LNF, Ngai D, Wang X, Kuriakose G, et al. Efferocytosis drives a tryptophan metabolism pathway in macrophages to promote tissue resolution. Nat Metab. 2024;6(9):1736–55. https://doi.org/10.1038/s42255/024–01115-7..
  - ⚬This work identified a tryptophan metabolism pathway activated during efferocytosis that promotes resolution and tissue repair, advancing understanding of metabolic-immune crosstalk.
- Patterson MT, Xu Y, Hillman H, Osinski V, Schrank PR, Kennedy AE, et al. Trem2 Agonist Reprograms Foamy Macrophages to Promote Atherosclerotic Plaque Stability-Brief Report. Arterioscler Thromb Vasc Biol. 2024;44(7):1646–57. 10.1161/ATVBAHA.124.320797.
  - ⚬This study demonstrated that pharmacologic activation of TREM2 reprograms foamy macrophages and stabilizes atherosclerotic plaques, providing preclinical proof-of-concept for TREM2-targeted therapy.

## Data Availability

No datasets were generated or analysed during the current study.
